# Guided supportive care may benefit from predicting cancer treatment-induced toxicity—a methodology paper on utilization of nomograms to predict severe oral mucositis, Part I

**DOI:** 10.1007/s00520-025-09691-4

**Published:** 2025-07-01

**Authors:** Poolakkad S. Satheeshkumar, Joel B. Epstein, Deepshikha Kewlani, Roberto Pili

**Affiliations:** 1 Department of Medicine, Division of Hematology and Oncology, University at Buffalo, Buffalo, NY 14203 USA; 2 City of Hope, Comprehensive Cancer Center, Duarte, CA 91010 USA; 3 Jacobs School of Medicine and Biomedical Sciences, University at Buffalo, Buffalo, NY 14203 USA

**Keywords:** Chemotherapy-induced oral ulcerative mucositis, Hematopoietic stem cell transplants, Nomogram, Risk prediction, Enhanced oral care

## Abstract

**Background:**

Patients undergoing hematopoietic stem cell transplantation (HSCT) should maintain oral hygiene to minimize mouth problems from the high-dose conditioning regimen. Utilizing risk prediction tools such as nomograms may be beneficial in predicting severe oral complications such as mucositis.

**Methods:**

A study was performed using the National Inpatient Samples Database 2018 to investigate individuals admitted to the hospital for autologous HSCT. The nomogram tool was employed to predict oral ulcerative mucositis (UM), utilizing a logistic regression model with the variables age, gender, race, total body irradiation (TBI), and fluid and electrolyte disorders (fed), and further we applied our findings in 2021 autologous HSCT cohorts and 2018 allogenic HSCT cohorts.

**Results:**

A total of 1560 patients who encountered UM were identified among 10,700 patients who underwent HSCT. The analysis showed that a 30-year-old White female patient who undergoes HSCT has a 28.2% risk of developing UM, 95%CI: 17.1–42.9%, who also undertook TBI, and had fed. On the other hand, a 20-year-old White female patient who undergoes HSCT has a risk score of 28.9% (95%CI: 17.6–46.0%), who also underwent TBI, and had fed. Additionally, Black female patient with TBI and fed of the same age of 20 or 30 who undergoes HSCT along with TBI has a risk score of 29.3% (95%CI: 17.3–45.1%), and 27.7%; 95%CI: 16.8–42.0%. The application in 2021 and 2018 cohorts showed similar trends, UM risk higher in allogenic cohorts with 47.2% risk (95%CI: 37.7–57.09%) in a White female, having weight loss and TBI.

**Conclusion:**

This report shows that younger age group, females, Whites, and those who are expected to receive TBI and having fed could be considered to be at a higher risk for developing severe mucositis. Thus, routine use of nomogram can help identify those requiring enhanced preventive oral care for reducing the oral complications such as UM.

**Supplementary Information:**

The online version contains supplementary material available at 10.1007/s00520-025-09691-4.

## Introduction

Oral mucositis is a debilitating condition that causes inflammation, swelling, and ulcerations of oral mucosa. It often develops as a side effect of treatments like radiation therapy for head and neck cancers, chemotherapy, chemoradiotherapy, and hematopoietic stem cell transplantation (HSCT) [1]. HSCT patients encounter high-dose conditioning regimens (chemotherapy alone or with radiation treatment) regimens for hematologic malignancies [2], resulting in an array of adverse events. When the submucosa is disrupted, normally harmless microorganisms residing in the healthy mucosa can penetrate the tissue, triggering an immune response driven by mononuclear infiltrating cells [3]. The condition arises due to damage to the rapidly dividing epithelial cells in the oral mucosa, leading to tissue breakdown and increased susceptibility to secondary infections [4]. Consequently, many oral adverse events are possible, and HSCT patients face significant challenges; these patients are subject to proper oral hygiene practices, including regular and multiple dental visits, to reduce the risk of these oral adverse events [5]. Further, evidence suggests that HSCT patients are highly vulnerable to additional oral complications due to infections (viral, fungal, and bacterial), dry mouth (xerostomia), and oral bleeding [6]. Severe cases of mucositis can necessitate opioid pain management, enteral or parenteral nutrition, and prolonged hospitalization, thereby increasing healthcare costs and resource utilization [7].

The incidence and severity of mucositis vary among HSCT patients, with several risk factors contributing to its development. Patient-related factors, including age, sex, race, and treatment-related factors such as total body irradiation (TBI) and chemotherapy regimens, influence susceptibility to mucositis [8]. Ensuring oral hygiene is vital for patients undergoing HSCT since it is essential in minimizing the anticipated oral complications resulting from the high-dose conditioning regimen [9]. Pretreatment dental disease evaluation and oral hygiene practices are crucial for HSCT recipients as they help minimize oral issues that may arise from myelosuppression and immunosuppression [10]. While cryotherapy, low-level laser therapy, and growth factors are employed for preventive care for oral mucositis risk, learning about risks early on is essential to create individualized prevention plans [11]. Nomograms, statistical models that predict outcomes for each patient, have become popular as useful clinical tools for evaluating patient outcomes.

By incorporating multiple risk factors into a visual, user-friendly format, nomograms facilitate evidence-based decision-making and personalized treatment planning [12]. Nomograms are valuable tools for predicting cancer adverse events in dental and medical oncology, leveraging claims data to enhance clinical decision-making. Nomograms’ easy-to-use graphical interface makes complicated statistical models easier to understand. Thus, nomograms help healthcare providers predict hazards like oral mucositis, osteonecrosis, or other types of toxicity without needing to be experts in statistics. By integrating diverse variables from claims data—such as age, sex, comorbidities, medication records, and treatment history (e.g., chemotherapy or radiation)—nomograms deliver individualized risk estimates [12]. This personalized approach provides tailored risk stratification that supports precise clinical interventions. In dentistry, nomograms shall guide preventive strategies, such as prophylaxis for oral complications, while in medicine, they inform treatment adjustments, like cardioprotective measures for chemotherapy patients, fostering multidisciplinary care. However, limited research has explored the development of a nomogram specifically for predicting ulcerative mucositis (UM) in HSCT recipients. This study aims to address this gap by developing a predictive nomogram to estimate the risk of HSCT-related mucositis using patient demographic and clinical factors.

## Methods

### Study design and data source

We utilized data obtained from the National Inpatient Samples (NIS) Database 2018 [12]. The dataset includes a sample of patients admitted to the hospital for autologous and allogenic HSCT. The NIS is by far the biggest and most accessible to the general public. All non-Federal short-term hospitals in the United States are represented by the NIS. It captures over 97% of the US population and is derived from the HCUP State Inpatient Databases (SID), which contain over 7 million hospital inpatient records from 47 states plus DC. This retrospective cross-sectional study made use of the 2018 National Inpatient Sample (NIS) database, which is a deidentified database that contains clinical data from a wide range of institutions across the United States. The data used for the 2018 NIS database’s discharge reports came from the Healthcare Cost and Utilization Project (HCUP) run by the AHRQ. The HCUP does not require that users obtain IRB review from their institutions; hence, it has not submitted to the Institutional Review Board (IRB) as it utilized publicly available data that was de-identified or comprised only a limited data set. Therefore, obtaining patient consent is not feasible. This study exclusively involved adults aged over 18 years, thereby excluding individuals under 18 years of age. Based on a 20% stratified sample of discharges, NIS 2018 accounts for 97% of all inpatient hospital discharges in the USA, excluding those from rehabilitation and long-term acute care facilities.

#### Study population

This study included adult patients (≥ 18 years) who underwent HSCT and were identified using the 2018 and 2021 National Inpatient Sample (NIS) database. Autologous and allogenic HSCT procedures were captured using ICD-10-PCS codes, and UM using ICD 10 CM codes. (supplementary file 1).

### Study measurements and statistical analysis

In the 2018 HSCT cohorts, patient- and clinical-level variables were added as covariates. Demographic characteristics, clinical variables, and hospital-related factors were evaluated in this study. The study took into account patient factors for risk prediction, age, sex (male = 0 or female = 1), race (White = 1, Black = 2, Hispanic = 3, Other = 4), total body irradiation (TBI, (yes = 1 or no = 0)), fluid and electrolyte disorders (fed, (yes = 1 or no = 0)). Continuous variables, including age and length of stay (LOS), were reported as mean (SD) and compared using *t* tests. Categorical variables, such as sex, race, total body irradiation (TBI), weight loss, and fed, were expressed as percentages and analyzed using the chi-square test. To reduce the impact of outliers, the geometric mean (The variables LOS and total charges that were not normally distributed were subjected to log transformation) was calculated and analyzed using *t* tests.

### Regression modeling and nomogram construction

A predictive nomogram was constructed to estimate the risk of HSCT-related ulcerative mucositis (UM) using a logistic regression model. The model incorporated key clinical variables, including patient age, gender, race, and exposure to TBI, fed, all of which were included as significant risk factors for UM in HSCT recipients. Regression models, such as the logistic regression model, underpin the outputs of the nomogram, and the efficacy of the tool depends on the model. This article, being a methodological research paper, will discuss the significance of risk prediction models such as nomograms and how to adjust critical parameters to enhance the efficacy of a nomogram in delivering improved oral care for patients at elevated risk of severe oral mucositis. In addition, we have investigated the clinical and patient characteristics; descriptive statistics were utilized in order to identify the demographic and clinical characteristics of each group (the patient characteristics included the age, sex, ethnicity, household income, patient location, co-morbidities, and weight loss, and clinical characteristic included length of stay, cost/total charge, and total body irradiation). Furthermore, we have implemented the nomogram development in the Autologous 2018 and additional in the 2021 inpatient database and the 2018 Allogenic inpatient database. The datasets 2018 allogenic cohort and 2021 autologous inpatient database are identical in its data characteristics and capturing process as all of them are pre-processed by the HCUP. Consequently, we have employed its applicability on external datasets and thus conducted external validation. While we have employed its application for external validation, we have not conducted statistical validation due to the absence of crucial variables in the datasets.

Advantage of our methodology is its compatibility with various external datasets, thereby enhancing its generalizability. They adapt to variations in patient demographics and coding methodologies by evaluating nomograms in diverse populations, healthcare systems nationwide, and treatment protocols. This ensures that claims data accurately represents the diversity of real life. A nomogram predicting oral issues might be enhanced across institutions to ensure its efficacy in dental practices. Concurrently, medical personnel can categorize systemic hazards into groups to deliver optimal supportive care. Nomograms can identify high-risk patients, optimize resource utilization, and improve results across several sectors due to their versatile applications. However, nomograms currently lack certain features that limit their immediate clinical adoption. Robust validation metrics, such as the concordance index, are often missing, hindering assessment of discriminative ability and risking overfitting claims data.

Additionally, seamless integration into electronic health records (EHRs) or clinical workflows remains challenging, as nomograms may not interface easily with existing systems, reducing accessibility for busy clinicians. Problems with claims data quality, such as missing clinical details or insufficient coding, might make predictions even less accurate, especially for complex dental outcomes like radiation-induced caries. Nomograms have a lot of potential to become important clinical tools with the right decision despite certain gaps. Using established validation processes will make predictions more reliable so they can be trusted in a variety of situations. Making interfaces and platforms that work with EHRs and are easy to use would make them more accessible and allow doctors to analyze risks in real time during patient consultations. Modern data processing techniques like machine learning imputation for missing claims data could improve accuracy. These improvements will make nomograms even more helpful, allowing dentists to control oral health concerns and doctors to provide better systemic treatment. For instance, a nomogram predicting chemotherapy-related mucositis could guide dental prophylaxis timing, while one forecasting cardiotoxicity could prompt early cardiology referrals.

Future advancements, such as real-time data integration from claims and clinical sources, will allow nomograms to update risk estimates, reflecting evolving patient profiles dynamically. Cloud-based platforms could facilitate cross-institutional collaboration, further validating nomograms across global datasets. By addressing current limitations, nomograms can transform into powerful tools for precision oncology, empowering clinicians to deliver data-driven, patient-centered care. Their ability to bridge dental and medical domains, optimize resources, and enhance outcomes position them as future cornerstones of multidisciplinary cancer management [12].

After accounting for the NIS’s complex sampling procedure, we used a survey-weighted statistical method to get original estimates of the US population. Since they did not follow a normal distribution, we presented the geometric mean after log-transforming total charges and LOS. For LOS of 0 days, a value of 0.0001 was imputed to prevent a negative log. Further, the study took into account patient factors, including patient location (urban/rural using a six-category urban–rural classification system), primary payer (Medicare, Medicaid, Private Insurance, Self-pay, No charge, or Other), and primary payer based on zip code (first to fourth quartile). Comorbidities were categorized using the Elixhauser comorbidity index [13]. The variables for the Elixhauser comorbidity index are available in the HCUP database. Burden of illness examined included length of stay (LOS), and total charges. All analyses were two-tailed, and statistical significance was established at *P* < 0.05. All statistical analyses were conducted utilizing R version 4.3.2 (R Foundation for Statistical Computing, Vienna, Austria).

## Results

The study cohort was stratified into two groups: patients who developed ulcerative mucositis (*n* = 1560) and those who did not (*n* = 9140). (Table 1). This nomograms serve as graphical representations of complex statistical models, allowing for individualized risk estimation by assigning weighted scores to each predictor variable. In this study, the nomogram was developed through a multistep process:

**Table 1:** Baseline characteristics of 2018 autologous hematopoietic stem cell transplant (HSCT) patients stratified with or without oral ulcerative mucositis

	HCST without oral ulcerative mucositis (weighted)	HCST with oral ulcerative mucositis (weighted)	*P* value
*n*	9140	1560	
AGE (mean (SD))	57.91 (12.66)	56.76 (12.61)	0.19
Sex (%)			0.006
Female	3,485 (38.1)	710 (45.5)	
RACE (%)			0.39
White	5785 (65.7)	985 (64.6)	
Black	1260 (14.3)	280 (18.4)	
Hispanic	955 (10.8)	135 (8.9)	
Asian and Other	810 (9.2)	125 (8.2)	
Median household income (based on current year)			0.82
0–25th percentile	1855 (20.6)	305 (19.8)	
26th to 50th percentile	2245 (24.9)	410 (26.6)	
51 st to 75th percentile	2345.0 (26.0)	420 (27.3)	
76th to 100th percentile	2565 (28.5)	405 (26.3)	
Expected primary payer (%)			0.09
Medicare	2865 (31.4)	415 (26.6)	
Medicaid	850 (9.3)	215 (13.8)	
Private Insurance	4995 (54.7)	865 (55.4)	
Other	425 (4.7)	65 (4.2)	
Patient location: NCHS urban–rural code (%)			0.08
“Central” counties of metro areas of ≥ 1 million population	2905 (31.9)	425 (27.4)	
“Fringe” counties of metro areas of ≥ 1 million population	2560 (28.1)	415 (26.8)	
Counties in metro areas of 250,000–999,999 population	1670 (18.4)	395 (25.5)	
Counties in metro areas of 50,000–249,999 population	730 (8.0)	135 (8.7)	
Micropolitan counties	710 (7.8)	140 (9.0)	
Not metropolitan	525 (5.8)	40 (2.6)	
Weight loss	1440 (15.8)	270 (17.3)	0.56
Fluid and electrolyte discrepancy	4180 (45.7)	930 (59.6)	0.001
Weighted Elixir score mean (SD)	6.86 (7.22)	8.12 (7.78)	0.04
Length of stay (Geometric mean)	16 days	18 days	0.001
Total charge (Geometric mean)	$202,842	$221,969	0.1
Total body irradiation (%)	520 (5.7)	100 (6.4)	0.6

*SD* standard deviation, *NCHS* National Center for Health Statistics, $ United States’ Dollar

All frequencies and percentages are weighted

A logistic regression analysis was performed to identify significant predictors of UM. The regression coefficients obtained from this model were then used to construct the nomogram. Each predictor variable was assigned a specific point value proportional to its regression coefficient, allowing for the calculation of an individualized risk score.

**Fig. 1:**
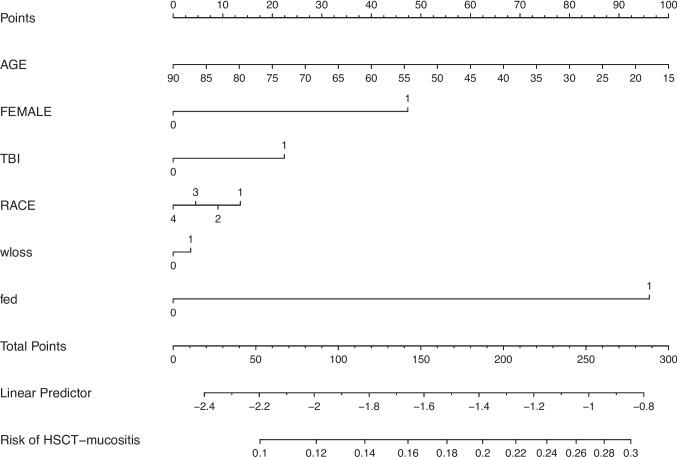
Nomogram constructed from Autologous 2018 model for predictingoralulcerative mucositis.** [**sex (male = 0 or female = 1), race (White = 1, Black = 2, Hispanic = 3, Asians and Others = 4), total body irradiation (TBI, (yes = 1 or no = 0)), fluid and electrolyte disorders (fed, (yes = 1 or no = 0)), weight loss (wloss, (yes = 1 or no = 0))**]**

The resulting nomogram provided individualized risk predictions for HSCT-related mucositis, with risk estimates ranging from 0.1 to 0.3 (Fig. 1). The mean age was similar between groups (57.54 ± 12.84 vs. 56.2 ± 13.62, *P* = 0.26). However, a significantly higher proportion of females developed mucositis (46.4% vs. 38.8%, *P* = 0.007). Race distribution did not significantly differ between the groups (*P* = 0.27), with White patients representing the majority in both cohorts. The geometric mean length of hospital stay was significantly longer in patients with mucositis (18.00 vs. 16 22 days, *P* = 0.004). However, the cost (total charge) was not significantly different in the two groups ($202,842 vs. $221,969, *P* = 0.1. TBI was slightly more frequent in the mucositis group (6.4% vs. 5.7%), though the difference was not statistically significant (*P* = 0.6). There were no significant differences in the incidence of weight loss between groups (17.3% s. 15.8%, *P* = 0.57). However, patients who developed ulcerative mucositis had a significantly higher rate of fed (59.6% vs. 45.7%, *P* = 0.001). These findings suggest that sex, length of hospital stay, total charge, and fluid/electrolyte imbalance may be associated with an increased risk of ulcerative mucositis in HSCT patients.

We developed the nomogram to predict the risk of oral ulcerative mucositis in patients undergoing hematopoietic stem cell transplantation (HSCT) based on key clinical factors. The model incorporates patient age, sex, total body irradiation (TBI), and race to generate a total risk score, which is then mapped to a linear predictor and correspond ng risk probability. Young age, female sex, and TBI exposure contribute to increased risk, while race has varying effects on the total risk score. The total points derived from these predictors are converted into a linear predictor value, which correlates with the estimated probability of developing HS T-related mucositis. This predictive tool provides a quantitative method for assessing mucositis risk, potentially aiding clinicians in patient counseling and risk stratification. The model showed age (Coefficient (Coeff)) of − 0.008, Standard error (SE) of 0.005, and *P* value of 0.09, females with Coeff: 0.28, and SE of 1.13, *P* value of 0.02; TBI with Coeff of 0.13, and SE of 0.26, *P* value of 0.59; Race with Coeff of − 0.27, and SE of 0.06, *P* value of 0.67; weight loss with Coeff of 0.02, and SE of 0.17, *P* value 0.89; fed with Coeff of 0.58, and SE of 0.13, *P* value of < 0.0001. The model showed brier score of 0.12, the model likelihood ratio was 29.5, with degree of freedom of 6, and P value for Chi sq < 0.0001. The model rank discrimination C score was 0.6, Somers’ Dxy of 0.2, gamma 0.2, and tau of 0.05.

The developed logistic regression model predicted the probability of the incidence of UM. The sample report (Fig. 1) labeled the outcome variable as “Risk of HSCT mucositis.” The tool is significant for inpatients undergoing HSCT treatment for liquid cancer. Utility of nomogram—the score can be calculated by the points corresponding to the age and the presence/absence of the other variables, and further total points correspond to the risk of HSCT mucositis. The risk can be predicted through Fig. 1/Table 2; a 20-year-old White female patient who undergoes HSCT has a risk score of 28.9% (95%CI: 17.6–46.0%), who also underwent TBI, and had fed. On the other hand, 30-year-old White female patient who undergoes HSCT has a 28.2% risk of developing UM, 95%CI: 17.1–42.9%, who also undertook TBI, and had fed (Fig. 1/Table 3). Otherwise, a 20-year-old Back male patient who undergoes HSCT has a risk score of 23.8% (95%CI: 13.7–38.0%), who also underwent TBI, and had fed, and this was 24.2% (95%CI: 13.9–38.9%) for 20-year-old White man with TBI and fed (Fig. 1/Table 2). For Hispanics female, who is 30 years old, the risk of HSCT mucositis with TBI and having fed was 27.2% (95%CI: 16.2–41.8%), and relatedly the risk was lower for 30-year-old female Asian and Others; this was 21.4% (95%CI: 12.1–35.2%) undergoing TBI and having fed. Consequently, HSCT patients who are younger, female, White, and anticipated to undergo TBI may be regarded as having an elevated risk for developing UM.

**Table 2:** Risk prediction tool examining patient aged 20 along with presence or absence of other variables

Patient numbers	Age	Female	Race	Weight loss	Fluid and electrolyte disbalance	Total body irradiation	Yhat (Effect estimate)	lower (confidence interval)	Upper (confidence interval)
1	20	No	White	No	No	No	0.1334595	0.0916682	0.1903116
2	20	Yes	White	No	No	No	0.1699395	0.11592629	0.2422237
3	20	No	Black	No	No	No	0.1303531	0.09103858	0.183223
4	20	Yes	Black	No	No	No	0.1661467	0.11517995	0.2337092
5	20	No	Hispanic	No	No	No	0.1273083	0.08730297	0.1819901
6	20	Yes	Hispanic	No	No	No	0.162422	0.11079424	0.2318348
7	20	No	Asians and Others	No	No	No	0.1243244	0.08114609	0.1858316
8	20	Yes	Asians and Others	No	No	No	0.1587649	0.10347509	0.2358265
9	20	No	White	Yes	No	No	0.1359287	0.08641149	0.2073802
10	20	Yes	White	Yes	No	No	0.1729489	0.11011323	0.2611199
11	20	No	Black	Yes	No	No	0.1327736	0.08567597	0.2000955
12	20	Yes	Black	Yes	No	No	0.1691026	0.10925423	0.252444
13	20	No	Hispanic	Yes	No	No	0.1296807	0.08250846	0.1980022
14	20	Yes	Hispanic	Yes	No	No	0.1653248	0.10545729	0.2496921
15	20	No	Asians and Others	Yes	No	No	0.1266493	0.07732778	0.2005901
16	20	Yes	Asians and Others	Yes	No	No	0.1616149	0.09917714	0.2523494
17	20	No	White	No	Yes	No	0.2153565	0.1526129	0.294918
18	20	Yes	White	No	Yes	No	0.2673165	0.1901655	0.3617848
19	20	No	Black	No	Yes	No	0.2108075	0.15110712	0.286144
20	20	Yes	Black	No	Yes	No	0.2620365	0.18846782	0.3518711
21	20	No	Hispanic	No	Yes	No	0.2063293	0.14482559	0.2852401
22	20	Yes	Hispanic	No	Yes	No	0.2568242	0.18129362	0.3503561
23	20	No	Asians and Others	No	Yes	No	0.2019219	0.13488845	0.2910601
24	20	Yes	Asians and Others	No	Yes	No	0.2516802	0.16976763	0.3561606
25	20	No	White	Yes	Yes	No	0.218958	0.14617203	0.3146319
26	20	Yes	White	Yes	Yes	No	0.2714863	0.18337084	0.3821305
27	20	No	Black	Yes	Yes	No	0.2143537	0.14464258	0.3056565
28	20	Yes	Black	Yes	Yes	No	0.2661538	0.18164611	0.3721006
29	20	No	Hispanic	Yes	Yes	No	0.2098202	0.13917605	0.3036728
30	20	Yes	Hispanic	Yes	Yes	No	0.2608886	0.17529217	0.3695528
31	20	No	Asians and Others	Yes	Yes	No	0.2053575	0.13051105	0.3079263
32	20	Yes	Asians and Others	Yes	Yes	No	0.2556912	0.16508004	0.3737727
33	20	No	White	No	No	Yes	0.1498308	0.08548097	0.2494117
34	20	Yes	White	No	No	Yes	0.1898055	0.10913934	0.3093872
35	20	No	Black	No	No	Yes	0.1464077	0.08424281	0.2423076
36	20	Yes	Black	No	No	Yes	0.1856685	0.10766036	0.3011256
37	20	No	Hispanic	No	No	Yes	0.1430496	0.08112291	0.2399067
38	20	Yes	Hispanic	No	No	Yes	0.1816015	0.10387953	0.2981284
39	20	No	Asians and Others	No	No	Yes	0.1397559	0.07642419	0.2418278
40	20	Yes	Asians and Others	No	No	Yes	0.1776041	0.098136	0.3000163
41	20	No	White	Yes	No	Yes	0.1525496	0.08233851	0.2653205
42	20	Yes	White	Yes	No	Yes	0.1930849	0.10556248	0.32667
43	20	No	Black	Yes	No	Yes	0.1490752	0.08115493	0.2578854
44	20	Yes	Black	Yes	No	Yes	0.1888931	0.10414278	0.3181213
45	20	No	Hispanic	Yes	No	Yes	0.1456663	0.07831344	0.2549241
46	20	Yes	Hispanic	Yes	No	Yes	0.1847714	0.10067184	0.3145536
47	20	No	Asians and Others	Yes	No	Yes	0.1423224	0.07404989	0.2561292
48	20	Yes	Asians and Others	Yes	No	Yes	0.1807197	0.09541807	0.3156677
49	20	No	White	No	Yes	Yes	0.2390031	0.14303387	0.371453
50	20	Yes	White	No	Yes	Yes	0.2945269	0.17984615	0.4428491
51	20	No	Black	No	Yes	Yes	0.2341036	0.14076715	0.3631697
52	20	Yes	Black	No	Yes	Yes	0.2889213	0.17722364	0.4338929
53	20	No	Hispanic	No	Yes	Yes	0.2292743	0.13555675	0.3607452
54	20	Yes	Hispanic	No	Yes	Yes	0.2833796	0.17109543	0.4310338
55	20	No	Asians and Others	No	Yes	Yes	0.2245155	0.1278881	0.3637037
56	20	Yes	Asians and Others	No	Yes	Yes	0.2779026	0.16198136	0.4338354
57	20	No	White	Yes	Yes	Yes	0.2428776	0.13932016	0.388651
58	20	Yes	White	Yes	Yes	Yes	0.2989479	0.17584181	0.4601229
59	20	No	Black	Yes	Yes	Yes	0.2379236	0.13716833	0.3800858
60	20	Yes	Black	Yes	Yes	Yes	0.2932932	0.17334202	0.4509677
61	20	No	Hispanic	Yes	Yes	Yes	0.2330395	0.13234777	0.3770479
62	20	Yes	Hispanic	Yes	Yes	Yes	0.2877016	0.16763247	0.4475323
63	20	No	Asians and Others	Yes	Yes	Yes	0.2282256	0.12525055	0.3791649
64	20	Yes	Asians and Others	Yes	Yes	Yes	0.2821739	0.15913821	0.4494847

**Table 3:** Risk prediction tool examining patient aged 30 along with presence or absence of other variables

Patient numbers	Age	Female	Race	Weight loss	Fluid and Electrolyte disbalance	Total body irradiation	Yhat (Effect Estimate)	Lower (confidence interval)	Upper (confidence interval)
1	30	No	White	No	No	No	0.124459	0.09121288	0.1675882
2	30	Yes	White	No	No	No	0.1589299	0.11524701	0.215144
3	30	No	Black	No	No	No	0.1215326	0.09056889	0.1612058
4	30	Yes	Black	No	No	No	0.1553368	0.11448419	0.2073538
5	30	No	Hispanic	No	No	No	0.1186657	0. 08619166	0.1612164
6	30	Yes	Hispanic	No	No	No	0.1518104	0.10940134	0.206841
7	30	No	Asian and Others	No	No	No	0.1158576	0. 07921969	0.1663781
8	30	Yes	Asian and Others	No	No	No	0.1483499	0.10115036	0.2123702
9	30	No	White	Yes	No	No	0.126786	0. 08479629	0.1853571
10	30	Yes	White	Yes	No	No	0.1617823	0.10817371	0.2349589
11	30	No	Black	Yes	No	No	0.1238126	0. 08398952	0.1788314
12	30	Yes	Black	Yes	No	No	0.1581369	0.10723705	0.2270519
13	30	No	Hispanic	Yes	No	No	0.1208994	0.08049139	0.1776732
14	30	Yes	Hispanic	Yes	No	No	0.1545585	0.10304742	0.2253495
15	30	No	Asian and Others	Yes	No	No	0.1180454	0.07489274	0.1811925
16	30	Yes	Asian and Others	Yes	No	No	0.1510465	0.09624954	0.229131
17	30	No	White	No	Yes	No	0.202121	0.15226551	0.2632317
18	30	Yes	White	No	Yes	No	0.2519129	0.18965562	0.3263759
19	30	No	Black	No	Yes	No	0.1977811	0.15063335	0.2552512
20	30	Yes	Black	No	Yes	No	0.2468346	0.18783289	0.3171329
21	30	No	Hispanic	No	Yes	No	0.1935118	0.1432416	0.2561496
22	30	Yes	Hispanic	No	Yes	No	0.2418256	0.17945719	0.3174829
23	30	No	Asian and Others	No	Yes	No	0.1893129	0.13196416	0.2640042
24	30	Yes	Asian and Others	No	Yes	No	0.2368863	0.16639833	0.3255718
25	30	No	White	Yes	Yes	No	0.2055592	0.14412444	0.284477
26	30	Yes	White	Yes	Yes	No	0.2559264	0.18105283	0.3485838
27	30	No	Black	Yes	Yes	No	0.2011641	0.14244086	0.2762967
28	30	Yes	Black	Yes	Yes	No	0.2507943	0.17916519	0.3392255
29	30	No	Hispanic	Yes	Yes	No	0.1968396	0.13633281	0.2756303
30	30	Yes	Hispanic	Yes	Yes	No	0.2457312	0.17206324	0.338061
31	30	No	Asian and Others	Yes	Yes	No	0.1925858	0.12687322	0.2813655
32	30	Yes	Asian and Others	Yes	Yes	No	0.2407374	0.16088049	0.3439838
33	30	No	White	No	No	Yes	0.1399044	0.08303599	0.226116
34	30	Yes	White	No	No	Yes	0.1777846	0.10618902	0.2824004
35	30	No	Black	No	No	Yes	0.1366716	0.08170142	0.2197752
36	30	Yes	Black	No	No	Yes	0.1738534	0.10459495	0.2748927
37	30	No	Hispanic	No	No	Yes	0.1335019	0.07840074	0.2181616
38	30	Yes	Hispanic	No	No	Yes	0.1699911	0.1005878	0.2727589
39	30	No	Asian and Others	No	No	Yes	0.1303946	0.07350808	0.2208122
40	30	Yes	Asian and Others	No	No	Yes	0.1661974	0.09458984	0.2755182
41	30	No	White	Yes	No	Yes	0.1424732	0.0796054	0.2419394
42	30	Yes	White	Yes	No	Yes	0.1809026	0.10225673	0.2998336
43	30	No	Black	Yes	No	Yes	0.1391907	0.07834135	0.2352397
44	30	Yes	Black	Yes	No	Yes	0.1769173	0.10073832	0.2919979
45	30	No	Hispanic	Yes	No	Yes	0.1359717	0.0753762	0.2330047
46	30	Yes	Hispanic	Yes	No	Yes	0.1730012	0.0971058	0.289214
47	30	No	Asian and Others	Yes	No	Yes	0.1328157	0.07099099	0.2348702
48	30	Yes	Asian and Others	Yes	No	Yes	0.169154	0.09168238	0.2911087
49	30	No	White	No	Yes	Yes	0.2247306	0.13938999	0.3415823
50	30	Yes	White	No	Yes	Yes	0.2781506	0.17562308	0.4107126
51	30	No	Black	No	Yes	Yes	0.2200391	0.13694501	0.334038
52	30	Yes	Black	No	Yes	Yes	0.2727362	0.17279116	0.4023711
53	30	No	Hispanic	No	Yes	Yes	0.2154183	0.13140614	0.3325765
54	30	Yes	Hispanic	No	Yes	Yes	0.2673882	0.16625685	0.4004867
55	30	No	Asian and Others	No	Yes	Yes	0.2108684	0.12337927	0.3365768
56	30	Yes	Asian and Others	No	Yes	Yes	0.2621072	0.15667756	0.4044566
57	30	No	White	Yes	Yes	Yes	0.2284432	0.1352281	0.359222
58	30	Yes	White	Yes	Yes	Yes	0.2824242	0.17108389	0.4287454
59	30	No	Black	Yes	Yes	Yes	0.2236966	0.13292573	0.3513361
60	30	Yes	Black	Yes	Yes	Yes	0.2769586	0.16840269	0.420138
61	30	No	Hispanic	Yes	Yes	Yes	0.2190206	0.12786086	0.3491541
62	30	Yes	Hispanic	Yes	Yes	Yes	0.2715587	0.16237958	0.4175524
63	30	No	Asian and Others	Yes	Yes	Yes	0.2144153	0.12050835	0.3521957
64	30	Yes	Asian and Others	Yes	Yes	Yes	0.2662253	0.1535372	0.4205308

**Fig. 2:**
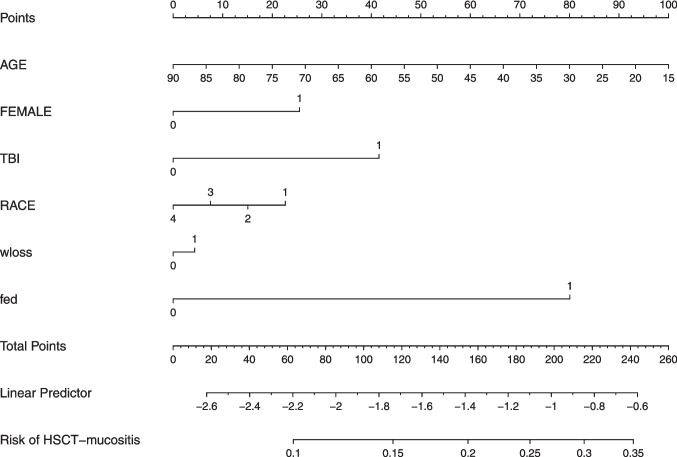
Nomogram constructed from Autologous 2021 model for predictingoralulcerative mucositis. [sex (male = 0 or female = 1), race (White = 1, Black = 2, Hispanic = 3, Asians and Others = 4), total body irradiation (TBI, (yes = 1 or no = 0)), fluid and electrolyte disorders (fed, (yes = 1 or no = 0)), weight loss (wloss, (yes = 1 or no = 0))**]**

**Fig. 3:**
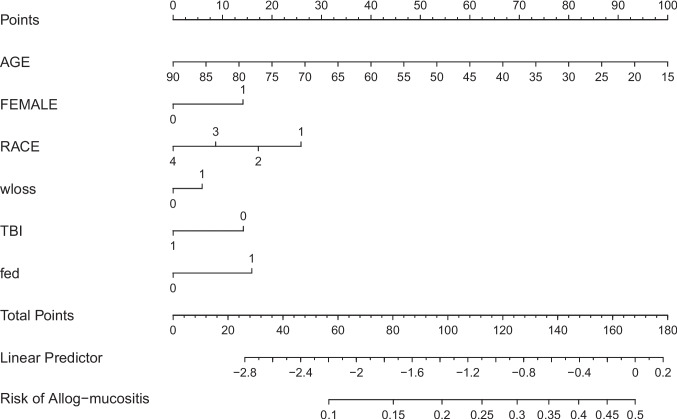
Nomogram constructed from Allogenic 2018 model for predicting oral ulcerative mucositis. **[**sex (male = 0 or female = 1), race (White = 1, Black = 2, Hispanic = 3, Asians and Others = 4), total body irradiation (TBI, (yes = 1 or no = 0)), fluid and electrolyte disorders (fed, (yes = 1 or no = 0)), weight loss (wloss, (yes = 1 or no = 0))**]**

Further we applied the nomogram development in the 2021 autologous cohorts (Fig. 2, Table 4), and in allogenic cohorts (Fig. 3, Table 5). The model coefficient, standard errors, odds ratio with 95% CI, and *P* values are provided in the supplementary file 2–4. Additionally, all three cohorts were analyzed for predictive assessment at the age of 25 years, revealing that females exposed to TBI, malnutrition, and weight loss exhibited a heightened susceptibility to mucositis, particularly in the 2018 allogenic cohort (supplementary file 5, 6, 7). A comparison analysis of all three cohorts within a young demographic—aged 25 years—revealed that female gender, regardless of racial group, exhibited an elevated risk of mucositis. Thus, age remains a crucial risk factor for oral mucositis, with mucositis risk increased in the nomogram in 2018 allogenic cohort up to 47.2% (95%CI: 37.7–57.09%) in a White female, having weight loss and TBI (Supplementary file 5).

**Table 4:** Baseline characteristics of 2021 autologous hematopoietic stem cell transplant (HSCT) patients stratified with or without oral ulcerative mucositis

	HCST without oral ulcerative mucositis (Weighted)	HCST with oral ulcerative mucositis (Weighted)	*P* value
*n*	9445	1470	
AGE (mean (SD))	57.71 (12.94)	56.03 (13.75)	0.090
Sex (%)			0.087
Female	3630 (38.4)	630 (42.9)	
RACE (%)			0.266
White	5710 (62.5)	>865 (59.7)	
Black	1420 (15.5)	225 (15.5)	
Hispanic	1235 (13.5)	155 (10.7)	
Asian and Other	770 (8.4)	205 (14.1)	
Median household income (based on current year)			0.468
0–25th percentile	2105 (22.7)	208 (19.2)	
26th to 50th percentile	1975 (21.3)	355 (24.4)	
51 st to 75th percentile	2620 (28.2)	385 (26.5)	
76th to 100th percentile	2590 (27.9)	435 (29.9)	
Expected primary payer (%)			0.390
Medicare	2715 (28.8)	500 (34.1)	
Medicaid	1065 (11.3)	160 (10.9)	
Private Insurance	5265 (55.8)	740 (50.5)	
Other	395 (4.2)	65 (4.4)	
Patient location: NCHS urban–rural code (%)			0.886
“Central” counties of metro areas of ≥ 1 million population	2840 (30.2)	475 (32.3)	
“Fringe” counties of metro areas of ≥ 1 million population	2690 (28.6)	420 (28.6)	
Counties in metro areas of 250,000–999,999 population	1870 (19.9)	295 (20.1)	
Counties in metro areas of 50,000–249,999 population	805 (8.6)	100 (6.8)	
Micropolitan counties	660 (7.0)	110 (7.5)	
Not metropolitan	540 (5.7)	70 (4.8)	
Weight loss	1680 (17.8)	335 (22.8)	0.112
Fluid and electrolyte discrepancy	4475 (47.4)	975 (66.3)	< 0.001
Weighted Elixir score mean (SD)	14.91 (7.1)	15.87 (7.45)	0.043
Length of stay (Geometric mean)	17.63 (7.23)	18.68 (5.73)	0.027
Total charge (Geometric mean)	$262,175	$288,152	0.379
Total body irradiation (%)	685 (7.3)	85 (5.8)	0.542

*SD* standard deviation, *NCHS* National Center for Health Statistics, $ United States’ Dollar

All frequencies and percentages are weighted

**Table 5:** Baseline characteristics of 2018 allogenic hematopoietic stem cell transplant (HSCT) patients stratified with or without oral ulcerative mucositis

	HCST without oral ulcerative mucositis (Weighted)	HCST with oral ulcerative mucositis (Weighted)	*P* value
*n*	5635	1515	
AGE (mean (SD))	53.68 (14.92)	48.21 (15.27)	< 0.001
Sex (%)			0.024
Female	2445 (43.4)	775 (51.2)	
RACE (%)			0.271
White	3825 (69.8)	1115 (74.6)	
Black	435 (7.9)	75 (5)	
Hispanic	555 (10.1)	170 (11.4)	
Asian and Other	665 (12.1)	135 (9)	
Median household income (based on current year)			0.361
0–25th percentile	980 (17.7)	250 (16.8)	
26th to 50th percentile	1310 (23.6)	390 (26.3)	
51 st to 75th percentile	1460 (26.3)	450 (30.3)	
76th to 100th percentile	1800 (32.4)	395 (26.6)	
Expected primary payer (%)			0.076
Medicare	1395 (24.8)	265 (17.5)	
Medicaid	670 (11.9)	220 (14.5)	
Private Insurance	3245 (57.6)	935 (61.7)	
Other	320 (5.7)	95 (6.3)	
Patient Location: NCHS Urban–Rural Code (%)			0.301
“Central” counties of metro areas of ≥ 1 million population	1725 (30.7)	460 (30.9)	
“Fringe” counties of metro areas of ≥ 1 million population	1645 (29.3)	345 (23.2)	
Counties in metro areas of 250,000–999,999 population	1100 (19.6)	340 (22.8)	
Counties in metro areas of 50,000–249,999 population	465 (8.3)	145 (9.7)	
Micropolitan counties	400 (7.1)	135 (9.1)	
Not metropolitan	280 (5)	65 (4.4)	
Weight loss	1255 (22.3)	380 (25.1)	0.433
Fluid and electrolyte discrepancy	2580 (45.8)	805 (53.1)	0.085
Weighted Elixir score mean (SD)	6.67 (7.41)	5.72 (6.32)	0.052
Length of stay (Geometric mean)	29.5 (18.95)	29.42 (12.33)	0.939
Total charge (Geometric mean)	$559,707	$549,631	0.763
Total body irradiation (%)	180 (3.2)	35 (2.3)	0.401

*SD* standard deviation, *NCHS* National Center for Health Statistics, $ United States’ Dollar

All frequencies and percentages are weighted

## Discussion

Using data from the National Inpatient Sample (NIS) database, we constructed a logistic regression-based model incorporating key variables such as age sex, race, and TBI. This tool may help clinicians identify high-risk patients early, allowing for tailored interventions to mitigate the burden of mucositis in HSCT recipients.

HSCT patients who are younger, females, Whites, and those who are expected to receive TBI could be considered to be at a higher risk for developing UM. Accordingly, developing a nomogram utilizing other variables, including the dose, regimen, number of cycles of the specific conditioning regimen, and dental status, can offer a superior utility as a nomogram in the application. Providing and planning regular dental hygiene visits/care or enhanced oral care, including photobiomodulation for those in high-risk groups thus, could reduce the severity of oral mucositis, thereby reducing the cost of care and reducing systemic complications.

We previously used machine learning methods to predict the mucositis among those receiving chemotherapy, the antineoplastic chemotherapy-induced pancytopenia, agranulocytosis due to cancer chemotherapy, fed, age, anemia due to chemotherapy, median household income, and depression were the most important variables predicting the mucositis [14]. Nevertheless, we did not employ significantly other important variables in this risk prediction model to avoid discrepancy in the model as this is an all-payer based claims database with inherent limitation, rendering the nomogram tool that incorporates only demographic characteristics and elevated risk variables documented in the dataset. However, we used real-world patient information to construct this nomogram; thus, this could be applied in any clinical setting for risk prediction for oral complications in HSCT. Cancer care settings extensively use nomograms due to their capacity for risk prediction in-hospital outcomes and survival [15–19]. Nomograms can continue to expand to include additional risk factors for more accurate prediction, such as prior history of mucositis with chemotherapy, medical comorbidities, current oral hygiene, dry mouth, and preexisting dental disease to provide risk factor identification that may be addressed before HSCT leading to reduction in oral and associated sys emic adverse events. This report offers an efficient method for routinely using nomograms for risk prediction in dental and oncology clinics. The goal is to evaluate the impact of dental care on preventing major oral issues, prognosis, and other health consequences with the tool development.

A randomized clinical experiment has shown that acute leukemia patients receiving extensive dental care experienced less severe and less unpleasant oral consequences than those receiving limited dental care, hence highlighting the efficacy of mucositis prevention through enhanced dental hygiene [20]. In cancer patients, undergoing HSCT many treatment modalities contributed to the prevention or reduction of mucositis severity including light therapy, cryotherapy, growth factors, and cytokine [21–24]. In the context of patient care, preventative interventions are extremely helpful, particularly when it comes to enhancing the quality of life of the patient. And now since dentists serve as the primary quality improvers by preventing cancer treatment-induced mucositis specifically in dental oncology settings [25], the risk prediction model might be very beneficial in mitigating for best care practices. Thus, the model takes into account patients who might benefit more from the enhanced oral care or preventive measures and streamline care. This study presents a predictive model for UM in patients undergoing HSCT, leveraging demographic and clinical factors to estimate individual risk. The findings highlight the significance of age, sex, race, fed, and TBI in predicting UM, aligning with existing literature that associates these factors with increased mucosal toxicity following high-dose condition ng regimens [26–28]. The developed nomogram provides a clinically applicable tool for risk stratification, allowing for early intervention and tailored preventive strategies. Further, the results, specifically related to the cost, and length of stay are consistent with previous research, which has demonstrated that chemotherapy and radiation therapy associated mucosal barrier, increasing susceptibility to higher burden [29].

One of the most notable findings of this study is the association between female sex and increased risk of UM, with 46.4% of affected patients being female compared to 38.8% in the unaffected cohort (*P* = 0.007). This is consistent with prior research suggesting that hormonal influences, differences in immune response, and genetic predispositions may contribute to heightened mucosal sensitivity in females undergo ng HSCT [5, 28, 30]. Furthermore, while race was statistically significant in the risk prediction model, variations in UM prevalence among racial groups underscore the need for further exploration into genetic, socioeconomic, and healthcare-related disparities that may influence mucositis risk and severity. TBI has long been associated with mucosal damage, even though, the difference in TBI exposure between UM and non-UM groups was not statistically significant (*P* = 0.6). This may be due to sample size limitations, variations in conditioning regimens, or the protective effects of modern radiotherapy techniques. However, the nomogram suggests that TBI contributes to an increased overall risk, particularly when combined with other high-risk factors such as younger age and female sex. Another key finding is the significantly longer hospital stay observed in patients with UM (18.00 vs. 16.22 days, *P* = 0.004), emphasizing the clinical and economic burden of this complication. The extended length of stay is likely attributable to the need for pain management, nutritional support, and infection control measures, further reinforcing the importance of early identification and intervention for high-risk patients. Previous studies have established that severe mucositis is associated with increased healthcare resource utilization, including extended hospitalization and higher treatment costs [7, 31]. Additionally, the strong association between UM and fed highlights the systemic impact of severe mucositis, as dehydration and metabolic disturbances often result from impaired oral intake and increased inflammatory response.

In order to identify patients who could benefit from more stringent oral hygiene procedures, preventative therapies, and attentive clinical monitoring, the created nomogram is a practical and useful tool for individual risk assessment. Compared to conventional clinical judgment alone, the nomogram improves risk estimation accuracy by combining numerous clinical indicators into a single prediction model. The clinical value and generalizability of this nomogram are further enhanced by applying it to bigger, multiple cohorts and multi-year data. The application of this predictive tool could facilitate early interventions—including enhanced oral hygiene protocols, cryotherapy, and the use of targeted muco-protective agents, as suggested in current clinical guidelines.

The main advantages of utilizing nomogram include applying the findings in multiple external datasets: Employing nomograms across several external datasets enhances their generalizability significantly. Using electronic data to anticipate adverse events in cancer patients has this major benefit. Nomograms can be used with a wide range of demographics and in a wide range of healthcare settings, showing that they can adjust to changes in demographics, treatment methods, and coding standards [32–37]. This wide range of uses makes it more useful in real life, such as when it can work with a wider range of patient profiles than models that just use one dataset. For instance, nomograms that use large claims databases like SEER can be improved across institutions, which will make them better at finding high-risk patients and making the best use of resources, which will lead to better clinical outcomes in cancer [32, 38–40].

A significant issue with neglecting the concordance index (C-index) to evaluate nomograms is the potential for overfitting, resulting in skewed performance assessments [41–43]. In the absence of C-index validation, which assesses a model’s ability to distinguish between two groups, it is challenging to evaluate the nomogram’s efficacy in differentiating between individuals experiencing adverse events and those who are not. This may result in overly optimistic projections that do not universally applicable, particularly when the claims data encompasses a diver e array of patients. Neglecting to validate a model may obscure its deficiencies, so diminishing its reliability for clinical application and heightening the likelihood of erroneous treatment recommendations [44–46]. As a result, this paper contributes as a methods paper to its application; however, we are very hopeful to apply the validation metrics for real-world application and utilization for supportive ca e in cancer cohorts. We are currently investigating the machine learning and artificial intelligence application with demographics, risk factors, that includes conditioning regimen, and other factors that are additionally determined through predictive analytics.

Nomograms utilize claims data to integrate many variables, including age, comorbidities, treatment history, and medication records, to establish a robust foundation for individualized risk assessments. Nomograms for breast cancer and colorectal cancer outcomes have demonstrated reliability in accurately representing complex patient characteristics [32, 40, 41]. The generalizability of nomograms across diverse external datasets enhances their reliability, since they may be used to various demographics and healthcare systems. Prior research methods utilizing SEER data and additional datasets has shown that performance remains constant across institutions, hence reducing the likelihood of model bias specific to a singular dataset [42, 44, 45]. This is advantageous as it is applicable in numerous contexts, analogous to claims data in the real world [32]. For clinical validation, numerous nomograms undergo internal testing through techniques such as bootstrap resampling, demonstrating their reliability within the originating cohort. Internal validation indicates that nomograms for lung cancer and ovarian cancer are effective [7, 13]. In our study, we implemented this across several contexts of supplementary datasets, demonstrating that performance remains consistent, hence diminishing the probability of model bias associated with a unique dataset, specifically comparing the 2018 autologous to the 2018 allogenic and 2021 autologous cohorts.

## Limitations

While this study provides important insights, several limitations should be acknowledged. First, the retrospective nature of the analysis introduces the potential for selection bias and residual confounding. Second, the study relied on administrative data from the National Inpatient Sample (NIS), which may lack granularity regarding specific chemotherapy regimens, supportive care measures, and severity grading of mucositis.

Additionally, while the nomogram incorporates key risk factors such as age, sex, race, and total body irradiation (TBI), it does not account for other important clinical variables that could influence mucositis risk. For instance, grafting-related factors, such as donor type, conditioning regimen, and immune response to the transplant and oral health status, may significantly impact mucosal toxicity and recovery. However, the nomogram serves as a valuable instrument for clinical risk evaluation; nevertheless, its relevance is contingent upon the specific characteristics of the patient and the advancement of the disease.

More comprehensive models that integrate supplementary clinical and treatment-related variables could improve predicted accuracy. We are currently concentrating on enhancing the model with these characteristics and validating it across various patient demographics and treatment environments.

## Conclusion

Supportive care specific nomograms for predicting cancer adverse events are an innovative approach. They provide precise, customized risk evaluations utilizing conventional electronic data; with intuitive graphical interface, from variables such as age, racial and ethnic, and treatment history, may offer a novel approach to risk identification. This facilitates customized therapies such as oral mucositis prophylaxis and for other cancer treatment-induced toxicities. Additionally, nomograms can be crucial for healthcare organizations to decrease costs through standardized validation, enhanced data processing, and seamless integration with electronic health records. Dynamic risk assessments tailored to patient profiles may further enhance flexibility through real-time data integration and cloud platforms. Nomograms have the potential to transform precision oncology by aiding supportive care in identifying high-risk patients, optimizing resource allocation, and enhancing interdisciplinary treatment.

## Supplementary Information

Below is the link to the electronic supplementary material.

ESM 1 (DOCX 155 KB)

ESM 2 (DOCX 20.7 KB)

ESM 3 (DOCX 20.4 KB)

ESM 4 (DOCX 21.1 KB)

ESM 5 (DOCX 24.7 KB)

ESM 6 (DOCX 24.5 KB)

ESM 7 (25.7 KB)

## Data Availability

No datasets were generated or analysed during the current study.
