# Supplementary material for: Guided supportive care may benefit from predicting cancer treatment-induced toxicity—a methodology paper on utilization of nomograms to predict severe oral mucositis, Part I
Source: Support Care Cancer. 2025 Jul 1;33(7):651. doi: 10.1007/s00520-025-09691-4 (PMC12213968; doi:10.1007/s00520-025-09691-4)
Supplement: Supplementary file 1 — (DOCX 155 KB) [file 520_2025_9691_MOESM1_ESM.docx]

ICD 10 CM codes

**Oral Ulcerative mucositis ICD Code (K1231)**

ICD 10 PCS codes

**Autologous bone marrow transplant codes**

| **30230AZ** | Transfusion of Embryonic Stem Cells into Peripheral Vein, Open Approach |
| --- | --- |
| **30230G0** | Transfusion of Autologous Bone Marrow into Peripheral Vein, Open Approach |
| **30230X0** | Transfusion of Autologous Cord Blood Stem Cells into Peripheral Vein, Open Approach |
| **30230Y0** | Transfusion of Autologous Hematopoietic Stem Cells into Peripheral Vein, Open Approach |
| **30240AZ** | Transfusion of Embryonic Stem Cells into Central Vein, Open Approach |
| **30240G0** | Transfusion of Autologous Bone Marrow into Central Vein, Open Approach |
| **30240X0** | Transfusion of Autologous Cord Blood Stem Cells into Central Vein, Open Approach |
| **30240Y0** | Transfusion of Autologous Hematopoietic Stem Cells into Central Vein, Open Approach |

| **30233AZ*** | Transfusion of Embryonic Stem Cells into Peripheral Vein, Percutaneous Approach |
| --- | --- |
| **30233G0*** | Transfusion of Autologous Bone Marrow into Peripheral Vein, Percutaneous Approach |
| **30233X0*** | Transfusion of Autologous Cord Blood Stem Cells into Peripheral Vein, Percutaneous Approach |
| **30233Y0*** | Transfusion of Autologous Hematopoietic Stem Cells into Peripheral Vein, Percutaneous Approach |
| **30243AZ*** | Transfusion of Embryonic Stem Cells into Central Vein, Percutaneous Approach |
| **30243G0*** | Transfusion of Autologous Bone Marrow into Central Vein, Percutaneous Approach |
| **30243X0*** | Transfusion of Autologous Cord Blood Stem Cells into Central Vein, Percutaneous Approach |
| **30243Y0*** | Transfusion of Autologous Hematopoietic Stem Cells into Central Vein, Percutaneous Approach |

ICD 10 PCS

**Allogenic bone marrow transplant codes**

| **30230G2** | Transfusion of Allogeneic Related Bone Marrow into Peripheral Vein, Open Approach |
| --- | --- |
| **30230G3** | Transfusion of Allogeneic Unrelated Bone Marrow into Peripheral Vein, Open Approach |
| **30230G4** | Transfusion of Allogeneic Unspecified Bone Marrow into Peripheral Vein, Open Approach |
| **30230X0** | Transfusion of Autologous Cord Blood Stem Cells into Peripheral Vein, Open Approach |
| **30230X2** | Transfusion of Allogeneic Related Cord Blood Stem Cells into Peripheral Vein, Open Approach |
| **30230X3** | Transfusion of Allogeneic Unrelated Cord Blood Stem Cells into Peripheral Vein, Open Approach |
| **30230X4** | Transfusion of Allogeneic Unspecified Cord Blood Stem Cells into Peripheral Vein, Open Approach |
| **30230Y2** | Transfusion of Allogeneic Related Hematopoietic Stem Cells into Peripheral Vein, Open Approach |
| **30230Y3** | Transfusion of Allogeneic Unrelated Hematopoietic Stem Cells into Peripheral Vein, Open Approach |
| **30230Y4** | Transfusion of Allogeneic Unspecified Hematopoietic Stem Cells into Peripheral Vein, Open Approach |
| **30233G2** | Transfusion of Allogeneic Related Bone Marrow into Peripheral Vein, Percutaneous Approach |
| **30233G3** | Transfusion of Allogeneic Unrelated Bone Marrow into Peripheral Vein, Percutaneous Approach |
| **30233G4** | Transfusion of Allogeneic Unspecified Bone Marrow into Peripheral Vein, Percutaneous Approach |
| **30233X2** | Transfusion of Allogeneic Related Cord Blood Stem Cells into Peripheral Vein, Percutaneous Approach |
| **30233X3** | Transfusion of Allogeneic Unrelated Cord Blood Stem Cells into Peripheral Vein, Percutaneous Approach |
| **30233X4** | Transfusion of Allogeneic Unspecified Cord Blood Stem Cells into Peripheral Vein, Percutaneous Approach |
| **30233Y2** | Transfusion of Allogeneic Related Hematopoietic Stem Cells into Peripheral Vein, Percutaneous Approach |
| **30233Y3** | Transfusion of Allogeneic Unrelated Hematopoietic Stem Cells into Peripheral Vein, Percutaneous Approach |
| **30233Y4** | Transfusion of Allogeneic Unspecified Hematopoietic Stem Cells into Peripheral Vein, Percutaneous Approach |
| **30240G2** | Transfusion of Allogeneic Related Bone Marrow into Central Vein, Open Approach |
| **30240G3** | Transfusion of Allogeneic Unrelated Bone Marrow into Central Vein, Open Approach |
| **30240G4** | Transfusion of Allogeneic Unspecified Bone Marrow into Central Vein, Open Approach |
| **30240X0** | Transfusion of Autologous Cord Blood Stem Cells into Central Vein, Open Approach |
| **30240X2** | Transfusion of Allogeneic Related Cord Blood Stem Cells into Central Vein, Open Approach |
| **30240X3** | Transfusion of Allogeneic Unrelated Cord Blood Stem Cells into Central Vein, Open Approach |
| **30240X4** | Transfusion of Allogeneic Unspecified Cord Blood Stem Cells into Central Vein, Open Approach |
| **30240Y2** | Transfusion of Allogeneic Related Hematopoietic Stem Cells into Central Vein, Open Approach |
| **30240Y3** | Transfusion of Allogeneic Unrelated Hematopoietic Stem Cells into Central Vein, Open Approach |
| **30240Y4** | Transfusion of Allogeneic Unspecified Hematopoietic Stem Cells into Central Vein, Open Approach |
| **30243G2** | Transfusion of Allogeneic Related Bone Marrow into Central Vein, Percutaneous Approach |
| **30243G3** | Transfusion of Allogeneic Unrelated Bone Marrow into Central Vein, Percutaneous Approach |
| **30243G4** | Transfusion of Allogeneic Unspecified Bone Marrow into Central Vein, Percutaneous Approach |
| **30243X2** | Transfusion of Allogeneic Related Cord Blood Stem Cells into Central Vein, Percutaneous Approach |
| **30243X3** | Transfusion of Allogeneic Unrelated Cord Blood Stem Cells into Central Vein, Percutaneous Approach |
| **30243X4** | Transfusion of Allogeneic Unspecified Cord Blood Stem Cells into Central Vein, Percutaneous Approach |
| **30243Y2** | Transfusion of Allogeneic Related Hematopoietic Stem Cells into Central Vein, Percutaneous Approach |
| **30243Y3** | Transfusion of Allogeneic Unrelated Hematopoietic Stem Cells into Central Vein, Percutaneous Approach |
| **30243Y4** | Transfusion of Allogeneic Unspecified Hematopoietic Stem Cells into Central Vein, Percutaneous Approach |
| **30250G1** | Transfusion of Nonautologous Bone Marrow into Peripheral Artery, Open Approach |
| **30250X0** | Transfusion of Autologous Cord Blood Stem Cells into Peripheral Artery, Open Approach |
| **30250X1** | Transfusion of Nonautologous Cord Blood Stem Cells into Peripheral Artery, Open Approach |
| **30250Y1** | Transfusion of Nonautologous Hematopoietic Stem Cells into Peripheral Artery, Open Approach |
| **30260G1** | Transfusion of Nonautologous Bone Marrow into Central Artery, Open Approach |
| **30260X0** | Transfusion of Autologous Cord Blood Stem Cells into Central Artery, Open Approach |
| **30260X1** | Transfusion of Nonautologous Cord Blood Stem Cells into Central Artery, Open Approach |
| **30260Y1** | Transfusion of Nonautologous Hematopoietic Stem Cells into Central Artery, Open Approach |
| **30253X1** | Transfusion of Nonautologous Cord Blood Stem Cells into Peripheral Artery, Percutaneous Approach |
| **30253G1** | Transfusion of Nonautologous Bone Marrow into Peripheral Artery, Percutaneous Approach |

**30263G1** Transfusion of Nonautologous Bone Marrow into Central Artery, Percutaneous Approach

**30263X1** Transfusion of Nonautologous Cord Blood Stem Cells into Central Artery, Percutaneous Approach

**30263Y1** Transfusion of Nonautologous Hematopoietic Stem Cells into Central Artery, Percutaneous Approach.

**Oral Ulcerative mucositis ICD Code (K1231)**
