# Supplementary material for: Guided supportive care may benefit from predicting cancer treatment-induced toxicity—a methodology paper on utilization of nomograms to predict severe oral mucositis, Part I
Source: Support Care Cancer. 2025 Jul 1;33(7):651. doi: 10.1007/s00520-025-09691-4 (PMC12213968; doi:10.1007/s00520-025-09691-4)
Supplement: Supplementary file 2 — (DOCX 20.7 KB) [file 520_2025_9691_MOESM2_ESM.docx]

> Model_2018_Autolog

Logistic Regression Model

**P(Y=1|X) = 1 / (1 + exp (- (β₀ + β₁X₁ + β₂X₂ + ... + βₙXₙ)))**

|  | **Model Likelihood Ratio Test** | **Discrimination**  **Indexes** | **Rank** | **Discrim.**  **Indexes** |
| --- | --- | --- | --- | --- |
| Obs 2067 | LR chi2 29.50 | R2 0.025 | C | 0.600 |
| 0 (Unweighted) 1762 | d.f. 6 | R2(6,2067)0.011 | Dxy | 0.201 |
| 1 (Unweighted) 305 | Pr (> chi2) <0.0001 | R2(6,780)0.030 | gamma | 0.201 |
| max lderivl 9e-11 |  | Brier 0.124 | tau-a | 0.051 |

|  | **Coef** | **S.E.** | **Wald Z** | **Pr(>IZI)** |
| --- | --- | --- | --- | --- |
| **Intercept** | -1.6833 | 0.3234 | -5.20 | <0.0001 |
| **AGE** | -0.0080 | 0.0048 | -1.65 | 0.0979 |
| **FEMALE** | 0.2847 | 0.1261 | 2.26 | 0.0240 |
| **TBI** | 0.1348 | 0.2551 | 0.53 | 0.5972 |
| **RACE** | -0.0271 | 0.0642 | -0.42 | 0.6728 |
| **wloss** | 0.0212 | 0.1676 | 0.13 | 0.8994 |
| **fed** | 0.5778 | 0.1272 | 4.54 | <0.0001 |

> summary (Model_2018_Autolog)

| **Factor** | **Low** | **High** | **Diff.** | **Effect** | **S.E.** | **Lower 0.95** | **Upper 0.95** |
| --- | --- | --- | --- | --- | --- | --- | --- |
| **AGE** | 52 | 67 | 15 | -0.120230 | 0.072649 | -0.262620 | 0.022156 |
| **Odds Ratio** |  |  |  | **0.886710** | **NA** | **0.769030** | **1.022400** |
| **FEMALE** | 0 | 1 | 1 | 0.284650 | 0.126100 | 0.037504 | 0.531800 |
| **Odds Ratio** | **0** | **1** | **1** | **1.329300** | **NA** | **1.038200** | **1.702000** |
| **TBI** | 0 | 1 | 1 | 0.134780 | 0.255050 | -0.365110 | 0.634680 |
| **Odds Ratio** | **0** | **1** | **1** | **1.144300** | **NA** | **0.694120** | **1.886400** |
| **RACE** | 1 | 2 | 1 | -0.027130 | 0.064249 | -0.153060 | 0.098795 |
| **Odds Ratio** | **1** | **2** | **1** | **0.973230** | **NA** | **0.858080** | **1.103800** |
| **wloss** | 0 | 1 | 1 | 0.021186 | 0.167620 | -0.307330 | 0.349710 |
| **Odds Ratio** | **0** | **1** | **1** | **1.021400** | **NA** | **0.735400** | **1.418600** |
| **fed** | 0 | 1 | 1 | 0.577780 | 0.127230 | 0.328420 | 0.827130 |
| **Odds Ratio** | **0** | **1** | **1** | **1.782100** | **NA** | **1.388800** | **2.286800** |
