# Supplementary material for: Guided supportive care may benefit from predicting cancer treatment-induced toxicity—a methodology paper on utilization of nomograms to predict severe oral mucositis, Part I
Source: Support Care Cancer. 2025 Jul 1;33(7):651. doi: 10.1007/s00520-025-09691-4 (PMC12213968; doi:10.1007/s00520-025-09691-4)
Supplement: Supplementary file 3 — (DOCX 20.4 KB) [file 520_2025_9691_MOESM3_ESM.docx]

> Model_2018_Allogenic

Logistic Regression Model

**P(Y=1|X) = 1 / (1 + exp (- (β₀ + β₁X₁ + β₂X₂ + ... + βₙXₙ)))**

|  | **Model Likelihood**  **Ratio Test** | **Discrimination**  **Indexes** | **Rank** | **Discrim.**  **Indexes** |
| --- | --- | --- | --- | --- |
| Obs 1394 | LR chi2 50.16 | R2 0.055 | C | 0.642 |
| 0 1095 | d.f. 6 | R2(6,1394)0.031 | Dxy | 0.285 |
| 1 299 | Pr(> chi2) **<0.0001** | R2(6,704.6)0.061 | gamma | 0.285 |
| max lderivl 2e-09 |  | Brier 0.163 | tau-a | 0.096 |

|  | **Coef** | **S.E.** | **Wald Z** | **Pr(>IZI)** |
| --- | --- | --- | --- | --- |
| **Intercept** | 0.0097 | 0.2825 | 0.03 | 0.9726 |
| **AGE** | -0.0263 | 0.0043 | -6.07 | <0.0001 |
| **FEMALE** | 0.2785 | 0.1333 | 2.09 | 0.0367 |
| **RACE** | -0.1699 | 0.0675 | -2.52 | 0.0119 |
| **wloss** | 0.1157 | 0.1564 | 0.74 | 0.4595 |
| **TBI** | -0.2801 | 0.4256 | -0.66 | 0.5105 |
| **fed** | 0.3137 | 0.1343 | 2.34 | 0.0195 |

> summary (Model_2018_Allogenic)

| Factor | Low | High | Diff. | Effect | S.E. | Lower 0.95 | Upper 0.95 |
| --- | --- | --- | --- | --- | --- | --- | --- |
| **AGE** | 42 | 64 | 22 | -0.57803 | 0.095270 | -0.764760 | -0.391300 |
| Odds Ratio |  |  |  | **0.56100** | **NA** | **0.465450** | **0.676170** |
| **FEMALE** | 0 | 1 | 1 | 0.27847 | 0.133320 | 0.017171 | 0.539760 |
| Odds Ratio | 0 | 1 | 1 | 1.32110 | **NA** | 1.017300 | 1.715600 |
| **RACE** | 1 | 2 | 1 | -0.16988 | 0.067514 | -0.302210 | -0.037555 |
| Odds Ratio | 1 | 2 | 1 | 0.84377 | **NA** | 0.739190 | 0.963140 |
| **wloss** | 0 | 1 | 1 | 0.11568 | 0.156400 | -0.190870 | 0.422220 |
| Odds Ratio | 0 | 1 | 1 | 1.12260 | **NA** | 0.826240 | 1.525300 |
| **TBI** | 0 | 1 | 1 | -0.28008 | 0.425590 | -1.114200 | 0.554070 |
| Odds Ratio | 0 | 1 | 1 | 0.75573 | **NA** | 0.328170 | 1.740300 |
| **fed** | 0 | 1 | 1 | 0.31370 | 0.134320 | 0.050440 | 0.576950 |
| Odds Ratio | 0 | 1 | 1 | 1.36850 | **NA** | 1.051700 | 1.780600 |
