# Supplementary material for: Guided supportive care may benefit from predicting cancer treatment-induced toxicity—a methodology paper on utilization of nomograms to predict severe oral mucositis, Part I
Source: Support Care Cancer. 2025 Jul 1;33(7):651. doi: 10.1007/s00520-025-09691-4 (PMC12213968; doi:10.1007/s00520-025-09691-4)
Supplement: Supplementary file 4 — (DOCX 21.1 KB) [file 520_2025_9691_MOESM4_ESM.docx]

> Model_2021_Autolog

Logistic Regression Model

**P(Y=1|X) = 1 / (1 + exp (- (β₀ + β₁X₁ + β₂X₂ + ... + βₙXₙ)))**

|  | Model Likelihood  Ratio Test | Discrimination  Indexes | Rank | Discrim.  Indexes |
| --- | --- | --- | --- | --- |
| Obs 1449 | LR chi2 31.43 | R2 0.037 | C | 0.620 |
| 0 1227 | d.f. 6 | R2(6,1449)0.017 | Dxy | 0.240 |
| 1 222 | Pr (> chi2) <0.0001 | R2(6,564)0.044 | gamma | 0.241 |
| max lderivl 2e-09 |  | Brier 0.127 | tau-a | 0.062 |

|  | Coef | S.E. | Wald Z | Pr(>IZI) |
| --- | --- | --- | --- | --- |
| Intercept | -1.4271 | 0.3788 | -3.77 | 0.0002 |
| AGE | -0.0118 | 0.0057 | -2.08 | 0.0372 |
| FEMALE | 0.2256 | 0.1488 | 1.52 | 0.1295 |
| TBI | 0.3675 | 0.2859 | 1.29 | 0.1986 |
| RACE | -0.0667 | 0.0770 | -0.87 | 0.3861 |
| wloss | 0.0382 | 0.1942 | 0.20 | 0.8440 |
| fed | 0.7082 | 0.1507 | 4.70 | <0.0001 |

> summary (Model_2021_Autolog)

| **Factors** | **Low** | **High** | **Diff.** | **Effect** | **Standard Error** | **Lower 0.95** | **Upper 0.95** |
| --- | --- | --- | --- | --- | --- | --- | --- |
| AGE | 52 | 67 | 15 | -0.176890 | 0.084883 | -0.343260 | -0.010522 |
| **Odds Ratio** |  |  |  | **0.837870** | **NA** | **0.709450** | **0.989530** |
| FEMALE | 0 | 1 | 1 | 0.225630 | 0.148840 | -0.066099 | 0.517350 |
| **Odds Ratio** | **0** | **1** | **1** | **1.253100** | **NA** | **0.936040** | **1.677600** |
| TBI | 0 | 1 | 1 | 0.367510 | 0.285860 | -0.192760 | 0.927770 |
| **Odds Ratio** | **0** | **1** | **1** | **1.444100** | **NA** | **0.824680** | **2.528900** |
| RACE | 1 | 2 | 1 | -0.066713 | 0.076975 | -0.217580 | 0.084155 |
| **Odds Ratio** | **1** | **2** | **1** | **0.935460** | **NA** | **0.804460** | **1.087800** |
| wloss | 0 | 1 | 1 | 0.038232 | 0.194240 | -0.342470 | 0.418940 |
| **Odds Ratio** | **0** | **1** | **1** | **1.039000** | **NA** | **0.710010** | **1.520300** |
| fed | 0 | 1 | 1 | 0.708190 | 0.150690 | 0.412850 | 1.003500 |
| **Odds Ratio** | **0** | **1** | **1** | **2.030300** | **NA** | **1.511100** | **2.727900** |
