# Supplementary material for: Guided supportive care may benefit from predicting cancer treatment-induced toxicity—a methodology paper on utilization of nomograms to predict severe oral mucositis, Part I
Source: Support Care Cancer. 2025 Jul 1;33(7):651. doi: 10.1007/s00520-025-09691-4 (PMC12213968; doi:10.1007/s00520-025-09691-4)
Supplement: Supplementary file 5 — (DOCX 24.7 KB) [file 520_2025_9691_MOESM5_ESM.docx]

**Table. Risk prediction tool examining patient aged 25 along with presence or absence of other variables in the 2018 Allogenic transplant cohort.**

| **Patient numbers** | **AGE** | **FEMALE** | **RACE** | **Weight loss** | **Fluid and electrolyte disbalance** | **Total body irradiation** | **Yhat (Effect estimate)** | **lower (Confidence Interval)** | **Upper (Confidence Interval)** |
| --- | --- | --- | --- | --- | --- | --- | --- | --- | --- |
| 1 | 25 | No | White | No | No | No | 0.3063927 | 0.24120315 | 0.3803689 |
| 2 | 25 | Yes | White | No | No | No | 0.3685196 | 0.29522138 | 0.448437 |
| 3 | 25 | No | Black | No | No | No | 0.2715211 | 0.21502653 | 0.3364959 |
| 4 | 25 | Yes | Black | No | No | No | 0.3299407 | 0.26580649 | 0.4010955 |
| 5 | 25 | No | Hispanic | No | No | No | 0.2392494 | 0.18272078 | 0.3067035 |
| 6 | 25 | Yes | Hispanic | No | No | No | 0.2935232 | 0.22858174 | 0.3681108 |
| 7 | 25 | No | Asians and Others | No | No | No | 0.2097092 | 0.14897532 | 0.2868571 |
| 8 | 25 | Yes | Asians and Others | No | No | No | 0.2595683 | 0.18858686 | 0.3458778 |
| 9 | 25 | No | White | Yes | No | No | 0.3315105 | 0.24927426 | 0.4254998 |
| 10 | 25 | Yes | White | Yes | No | No | 0.395824 | 0.30347168 | 0.4962572 |
| 11 | 25 | No | Black | Yes | No | No | 0.2949961 | 0.22134823 | 0.3811533 |
| 12 | 25 | Yes | Black | Yes | No | No | 0.3559982 | 0.27210794 | 0.4497705 |
| 13 | 25 | No | Hispanic | Yes | No | No | 0.2609336 | 0.18891554 | 0.3486063 |
| 14 | 25 | Yes | Hispanic | Yes | No | No | 0.3180699 | 0.23494841 | 0.4146598 |
| 15 | 25 | No | Asians and Others | Yes | No | No | 0.2295239 | 0.15547994 | 0.3252492 |
| 16 | 25 | Yes | Asians and Others | Yes | No | No | 0.2824104 | 0.19568424 | 0.3889845 |
| 17 | 25 | No | White | No | Yes | No | 0.2502808 | 0.12113065 | 0.4470828 |
| 18 | 25 | Yes | White | No | Yes | No | 0.3060503 | 0.15558878 | 0.5135274 |
| 19 | 25 | No | Black | No | Yes | No | 0.219772 | 0.10525032 | 0.4028055 |
| 20 | 25 | Yes | Black | No | Yes | No | 0.2712025 | 0.13603964 | 0.4679241 |
| 21 | 25 | No | Hispanic | No | Yes | No | 0.1920296 | 0.08959626 | 0.3646641 |
| 22 | 25 | Yes | Hispanic | No | Yes | No | 0.2389562 | 0.11650305 | 0.4277957 |
| 23 | 25 | No | Asians and Others | No | Yes | No | 0.1670395 | 0.07472165 | 0.3324361 |
| 24 | 25 | Yes | Asians and Others | No | Yes | No | 0.2094423 | 0.09769666 | 0.3932914 |
| 25 | 25 | No | White | Yes | Yes | No | 0.2726065 | 0.12989738 | 0.4847522 |
| 26 | 25 | Yes | White | Yes | Yes | No | 0.3311535 | 0.1660209 | 0.5518502 |
| 27 | 25 | No | Black | Yes | Yes | No | 0.2402484 | 0.11286147 | 0.44009 |
| 28 | 25 | Yes | Black | Yes | Yes | No | 0.294661 | 0.14518266 | 0.5067969 |
| 29 | 25 | No | Hispanic | Yes | Yes | No | 0.210619 | 0.0961335 | 0.4009632 |
| 30 | 25 | Yes | Hispanic | Yes | Yes | No | 0.260623 | 0.12443955 | 0.466445 |
| 31 | 25 | No | Asians and Others | Yes | Yes | No | 0.1837599 | 0.0802748 | 0.3673649 |
| 32 | 25 | Yes | Asians and Others | Yes | Yes | No | 0.229239 | 0.10451559 | 0.4311409 |
| 33 | 25 | No | White | No | No | Yes | 0.3767554 | 0.29980764 | 0.4604647 |
| 34 | 25 | Yes | White | No | No | Yes | 0.4440163 | 0.36130605 | 0.5299503 |
| 35 | 25 | No | Black | No | No | Yes | 0.3377755 | 0.26932423 | 0.4137723 |
| 36 | 25 | Yes | Black | No | No | Yes | 0.4025725 | 0.32815231 | 0.4817677 |
| 37 | 25 | No | Hispanic | No | No | Yes | 0.3008816 | 0.23154037 | 0.3807012 |
| 38 | 25 | Yes | Hispanic | No | No | Yes | 0.362475 | 0.28584115 | 0.4468004 |
| 39 | 25 | No | Asians and Others | No | No | Yes | 0.2663964 | 0.19129394 | 0.3579339 |
| 40 | 25 | Yes | Asians and Others | No | No | Yes | 0.324204 | 0.2393374 | 0.4224508 |
| 41 | 25 | No | White | Yes | No | Yes | 0.4042794 | 0.31486847 | 0.5005311 |
| 42 | 25 | Yes | White | Yes | No | Yes | 0.472727 | 0.376641 | 0.5708768 |
| 43 | 25 | No | Black | Yes | No | Yes | 0.3641155 | 0.2824438 | 0.4544466 |
| 44 | 25 | Yes | Black | Yes | No | Yes | 0.4306792 | 0.34155116 | 0.5245374 |
| 45 | 25 | No | Hispanic | Yes | No | Yes | 0.3257598 | 0.24370286 | 0.4200989 |
| 46 | 25 | Yes | Hispanic | Yes | No | Yes | 0.3896079 | 0.29860623 | 0.4890074 |
| 47 | 25 | No | Asians and Others | Yes | No | Yes | 0.2896044 | 0.20275139 | 0.3952190 |
| 48 | 25 | Yes | Asians and Others | Yes | No | Yes | 0.3500451 | 0.25188061 | 0.4628003 |
| 49 | 25 | No | White | No | Yes | Yes | 0.3135833 | 0.15728588 | 0.5279017 |
| 50 | 25 | Yes | White | No | Yes | Yes | 0.376377 | 0.19982393 | 0.5932666 |
| 51 | 25 | No | Black | No | Yes | Yes | 0.2782216 | 0.13738978 | 0.4826421 |
| 52 | 25 | Yes | Black | No | Yes | Yes | 0.3374151 | 0.17585298 | 0.5486038 |
| 53 | 25 | No | Hispanic | No | Yes | Yes | 0.2454218 | 0.11759358 | 0.4425196 |
| 54 | 25 | Yes | Hispanic | No | Yes | Yes | 0.3005427 | 0.15161206 | 0.5081451 |
| 55 | 25 | No | Asians and Others | No | Yes | Yes | 0.2153352 | 0.098602 | 0.4077522 |
| 56 | 25 | Yes | Asians and Others | No | Yes | Yes | 0.2660816 | 0.12799109 | 0.4724402 |
| 57 | 25 | No | White | Yes | Yes | Yes | 0.3390025 | 0.16971415 | 0.5627103 |
| 58 | 25 | Yes | White | Yes | Yes | Yes | 0.4038913 | 0.21434327 | 0.6272382 |
| 59 | 25 | No | Black | Yes | Yes | Yes | 0.3020357 | 0.14834653 | 0.5180879 |
| 60 | 25 | Yes | Black | Yes | Yes | Yes | 0.3637424 | 0.18881476 | 0.5840485 |
| 61 | 25 | No | Hispanic | Yes | Yes | Yes | 0.2674688 | 0.12711142 | 0.4779488 |
| 62 | 25 | Yes | Hispanic | Yes | Yes | Yes | 0.325406 | 0.1630239 | 0.5443391 |
| 63 | 25 | No | Asians and Others | Yes | Yes | Yes | 0.235523 | 0.10674396 | 0.4426717 |
| 64 | 25 | Yes | Asians and Others | Yes | Yes | Yes | 0.289273 | 0.13788944 | 0.5087731 |
