# Supplementary material for: Guided supportive care may benefit from predicting cancer treatment-induced toxicity—a methodology paper on utilization of nomograms to predict severe oral mucositis, Part I
Source: Support Care Cancer. 2025 Jul 1;33(7):651. doi: 10.1007/s00520-025-09691-4 (PMC12213968; doi:10.1007/s00520-025-09691-4)
Supplement: Supplementary file 6 — (DOCX 24.5 KB) [file 520_2025_9691_MOESM6_ESM.docx]

**Table. Risk prediction tool examining patient aged 25 along with presence or absence of other variables in the 2021 Autologous transplant cohort.**

| **Patient numbers** | **AGE** | **FEMALE** | **RACE** | **Weight loss** | **Fluid and electrolyte disbalance** | **Total body irradiation** | **Yhat (Effect estimate)** | **lower (Confidence Interval)** | **Upper (Confidence Interval)** |
| --- | --- | --- | --- | --- | --- | --- | --- | --- | --- |
| 1 | 25 | No | White | No | No | No | 0.1288923 | 0.09153302 | 0.1785038 |
| 2 | 25 | Yes | White | No | No | No | 0.1643607 | 0.11569918 | 0.2282068 |
| 3 | 25 | No | Black | No | No | No | 0.1258767 | 0.0909014 | 0.1717665 |
| 4 | 25 | Yes | Black | No | No | No | 0.1606683 | 0.11495061 | 0.2200479 |
| 5 | 25 | No | Hispanic | No | No | No | 0.1229217 | 0.08686408 | 0.1711417 |
| 6 | 25 | Yes | Hispanic | No | No | No | 0.1570432 | 0.11023568 | 0.2188376 |
| 7 | 25 | No | Asians and Others | No | No | No | 0.1200265 | 0.08030335 | 0.1756466 |
| 8 | 25 | Yes | Asians and Others | No | No | No | 0.1534851 | 0.10245565 | 0.2235986 |
| 9 | 25 | No | White | Yes | No | No | 0.1312898 | 0.08571237 | 0.1959098 |
| 10 | 25 | Yes | White | Yes | No | No | 0.1672912 | 0.10927229 | 0.2475540 |
| 11 | 25 | No | Black | Yes | No | No | 0.1282264 | 0.08494683 | 0.1890019 |
| 12 | 25 | Yes | Black | Yes | No | No | 0.1635458 | 0.10838098 | 0.2392546 |
| 13 | 25 | No | Hispanic | Yes | No | No | 0.1252241 | 0.0816154 | 0.1873796 |
| 14 | 25 | Yes | Hispanic | Yes | No | No | 0.1598682 | 0.10439031 | 0.2370268 |
| 15 | 25 | No | Asians and Others | Yes | No | No | 0.1222822 | 0.07621763 | 0.1904479 |
| 16 | 25 | Yes | Asians and Others | Yes | No | No | 0.1562579 | 0.09784363 | 0.2402589 |
| 17 | 25 | No | White | No | Yes | No | 0.144797 | 0.08435594 | 0.2373199 |
| 18 | 25 | Yes | White | No | Yes | No | 0.1837189 | 0.10778194 | 0.2954409 |
| 19 | 25 | No | Black | No | Yes | No | 0.1414697 | 0.08307171 | 0.2305962 |
| 20 | 25 | Yes | Black | No | Yes | No | 0.1796851 | 0.10624825 | 0.2875497 |
| 21 | 25 | No | Hispanic | No | Yes | No | 0.1382065 | 0.07985808 | 0.2285959 |
| 22 | 25 | Yes | Hispanic | No | Yes | No | 0.1757209 | 0.10235109 | 0.2849875 |
| 23 | 25 | No | Asians and Others | No | Yes | No | 0.1350068 | 0.07505321 | 0.2308967 |
| 24 | 25 | Yes | Asians and Others | No | Yes | No | 0.1718258 | 0.09647044 | 0.2873247 |
| 25 | 25 | No | White | Yes | Yes | No | 0.1474402 | 0.08105984 | 0.2532019 |
| 26 | 25 | Yes | White | Yes | Yes | No | 0.1869174 | 0.10401747 | 0.3128196 |
| 27 | 25 | No | Black | Yes | Yes | No | 0.1440624 | 0.07983729 | 0.2461334 |
| 28 | 25 | Yes | Black | Yes | Yes | No | 0.1828291 | 0.10255027 | 0.3046209 |
| 29 | 25 | No | Hispanic | Yes | Yes | No | 0.1407492 | 0.07693044 | 0.2435417 |
| 30 | 25 | Yes | Hispanic | Yes | Yes | No | 0.1788106 | 0.09899494 | 0.3014482 |
| 31 | 25 | No | Asians and Others | Yes | Yes | No | 0.1375 | 0.07259783 | 0.2450905 |
| 32 | 25 | Yes | Asians and Others | Yes | Yes | No | 0.1748615 | 0.09364718 | 0.3029648 |
| 33 | 25 | No | White | No | No | Yes | 0.2086615 | 0.15256639 | 0.2786009 |
| 34 | 25 | Yes | White | No | No | Yes | 0.2595405 | 0.19005917 | 0.3436454 |
| 35 | 25 | No | Black | No | No | Yes | 0.2042171 | 0.1510082 | 0.2702075 |
| 36 | 25 | Yes | Black | No | No | Yes | 0.2543607 | 0.18831021 | 0.3340443 |
| 37 | 25 | No | Hispanic | No | No | Yes | 0.1998435 | 0.14420704 | 0.2701685 |
| 38 | 25 | Yes | Hispanic | No | No | Yes | 0.2492495 | 0.18057278 | 0.3334181 |
| 39 | 25 | No | Asians and Others | No | No | Yes | 0.1955405 | 0.13360991 | 0.2769992 |
| 40 | 25 | Yes | Asians and Others | No | No | Yes | 0.2442074 | 0.16829515 | 0.3403489 |
| 41 | 25 | No | White | Yes | No | Yes | 0.2121813 | 0.14531228 | 0.2990549 |
| 42 | 25 | Yes | White | Yes | No | Yes | 0.2636326 | 0.1823993 | 0.3648976 |
| 43 | 25 | No | Black | Yes | No | Yes | 0.2076816 | 0.14371506 | 0.2904615 |
| 44 | 25 | Yes | Black | Yes | No | Yes | 0.2583997 | 0.18060368 | 0.3551809 |
| 45 | 25 | No | Hispanic | Yes | No | Yes | 0.2032527 | 0.13793428 | 0.2891285 |
| 46 | 25 | Yes | Hispanic | Yes | No | Yes | 0.14504519 | 0.07758975 | 0.2549371 |
| 47 | 25 | No | Asians and Others | Yes | No | Yes | 0.13964164 | 0.07446545 | 0.2466605 |
| 48 | 25 | Yes | Asians and Others | Yes | No | Yes | 0.1589641 | 0.08405315 | 0.2802135 |
| 49 | 25 | No | White | No | Yes | Yes | 0.16603484 | 0.09526313 | 0.2734901 |
| 50 | 25 | Yes | White | No | Yes | Yes | 0.18821045 | 0.10690157 | 0.3099031 |
| 51 | 25 | No | Black | No | Yes | Yes | 0.18154027 | 0.10649294 | 0.2921804 |
| 52 | 25 | Yes | Black | No | Yes | Yes | 0.20527704 | 0.11944462 | 0.3296947 |
| 53 | 25 | No | Hispanic | No | Yes | Yes | 0.19814967 | 0.11648208 | 0.3165603 |
| 54 | 25 | Yes | Hispanic | No | Yes | Yes | 0.2234652 | 0.13069753 | 0.3551755 |
| 55 | 25 | No | Asians and Others | No | Yes | Yes | 0.21587786 | 0.12482106 | 0.3470216 |
| 56 | 25 | Yes | Asians and Others | No | Yes | Yes | 0.24277259 | 0.14020392 | 0.3866349 |
| 57 | 25 | No | White | Yes | Yes | Yes | 0.20880003 | 0.1174834 | 0.3434704 |
| 58 | 25 | Yes | White | Yes | Yes | Yes | 0.23507735 | 0.13144607 | 0.3842643 |
| 59 | 25 | No | Black | Yes | Yes | Yes | 0.2272111 | 0.13101611 | 0.3644159 |
| 60 | 25 | Yes | Black | Yes | Yes | Yes | 0.25505849 | 0.14645359 | 0.4059016 |
| 61 | 25 | No | Hispanic | Yes | Yes | Yes | 0.2467393 | 0.14320185 | 0.3909761 |
| **62** | **25** | **Yes** | **Hispanic** | **Yes** | **Yes** | **Yes** | **0.27612492** | **0.16005216** | **0.432983** |
| 63 | 25 | No | Asians and Others | Yes | Yes | Yes | 0.26736521 | 0.15357267 | 0.4233068 |
| **64** | **25** | **Yes** | **Asians and Others** | **Yes** | **Yes** | **Yes** | **0.29823472** | **0.17173183** | **0.4655463** |
